# Deciphering Common Long QT Syndrome Using CRISPR/Cas9 in Human-Induced Pluripotent Stem Cell-Derived Cardiomyocytes

**DOI:** 10.3389/fcvm.2022.889519

**Published:** 2022-05-13

**Authors:** Yongfei Song, Zequn Zheng, Jiangfang Lian

**Affiliations:** ^1^Department of Cardiovascular, Ningbo Institute of Innovation for Combined Medicine and Engineering, Ningbo, China; ^2^Department of Cardiovascular, Medical College, Ningbo University, Ningbo, China; ^3^Department of Cardiovascular, Lihuili Hospital Affiliated to Ningbo University, Ningbo, China

**Keywords:** LQTS, hiPSC, hiPSC-CMs, CRISPR/Cas9, modifier genes, variants of uncertain significance (VUS)

## Abstract

From carrying potentially pathogenic genes to severe clinical phenotypes, the basic research in the inherited cardiac ion channel disease such as long QT syndrome (LQTS) has been a significant challenge in explaining gene-phenotype heterogeneity. These have opened up new pathways following the parallel development and successful application of stem cell and genome editing technologies. Stem cell-derived cardiomyocytes and subsequent genome editing have allowed researchers to introduce desired genes into cells in a dish to replicate the disease features of LQTS or replace causative genes to normalize the cellular phenotype. Importantly, this has made it possible to elucidate potential genetic modifiers contributing to clinical heterogeneity and hierarchically manage newly identified variants of uncertain significance (VUS) and more therapeutic options to be tested *in vitro*. In this paper, we focus on and summarize the recent advanced application of human-induced pluripotent stem cell-derived cardiomyocytes (hiPSC-CMs) combined with clustered regularly interspaced short palindromic repeats/CRISPR-associated system 9 (CRISPR/Cas9) in the interpretation for the gene-phenotype relationship of the common LQTS and presence challenges, increasing our understanding of the effects of mutations and the physiopathological mechanisms in the field of cardiac arrhythmias.

## Introduction

Long QT syndrome (LQTS) is an inherited cardiac channelopathy (ICCs) associated with prolonged cardiac repolarization caused by functional changes in cardiac ion channels. It is characterized by the prolongation of QT interval and fatal arrhythmias (1). Causative mutations with autosomal dominant inheritance are associated with congenital long QT syndrome in at least 1:2,000 live births, with type 1 LQTS (LQT1) to type 3 LQTS (LQT3) accounting for ~75% of clinically defined cases. *KCNQ1, KCNH2*, and *SCN5A* encode ion channels Kv7.1, Kv11.1, and Nav1.5 to cause LQT1, LQT2, and LQT3, respectively (2–5). Single nucleotide variants (SNVs) occurring in each gene to cause loss-of-function (LOF) (Kv7.1, Kv11.1) and gain-of-function (Nav1.5) to abnormal ionic currents in each channel are thought to underlie the pathogenesis of these ICCs (6).

The pillars of current therapy include the following: β-blockers (propranolol and nadolol), left cardiac sympathetic denervation, and the automatic implantable cardioverter-defibrillator (7). Various limitations, including drug intolerance, poor compliance, and high complications of invasive strategies, urgently call for safer and more effective drug administration (8). From correction of pathogenic phenotypes in the laboratory to clinical translation, an available treatment always requires robust disease models that ideally replicate the pathophysiological features of the patient without gross deviations.

Unfortunately, the search for pathogenesis and effective therapeutic strategies for ICC is limited mainly by the availability of disease models; while human primary cardiomyocytes would be an ideal option, their limited provenance, non-replication, and poor consistency hinder this choice. In desperation, animal models and various modified immortalized tool cells are always alternatives. Overexpression of mutant channels in heterologous expression systems, such as Xenopus oocytes, human embryonic kidney (HEK) cells, and Chinese hamster ovary (CHO) cells, has been used in most functional studies of specific mutations associated with LQTS. One significant limit of these models is that they lack critical cardiac ion channel macromolecular complex components that may be required to recapitulate the exact molecular and electrophysiological phenotype associated with the mutation (9, 10).

The rise of the human-induced pluripotent stem (hiPSC) technology and improvements in hiPSC directed differentiation schemes with CRISPR/Cas9 gene-editing tools have established powerful approaches for ICC-related research, human disease modeling, and drug development or screening (9). Cardiomyocytes generated by disease-specific hiPSC-induced differentiation called hiPSC-CMs are expected to retain a gene-specific clinical phenotype *in vitro* to better model disease (11). Furthermore, CRISPR/Cas9 enables us to create any variant we desire in hiPSC in a dish to generate isogenic cardiomyocyte lines. These isogenic hiPSC-CMs are generating new study ideas for SNV in LQTS, particularly for those classified as variations of unknown significance (VUS) that haven't been clinically defined and characterized in the lab, or even pathogenic variants to investigate the influence of modifier genes (7, 12, 13). More importantly, through the continuous development of improved CRISPR/Cas9, building on the successful modeling of hiPSC-CMs, we are able to use it to introduce or correct disease-causing mutations at a much smaller cost to the cell (14–16). In this paper, we will discuss how these technologies can be used to elucidate common LQTS-related gene phenotypes in a single dish and strategies for doing so, current achievements, and future challenges.

## hiPSC-CMs in LQTS

### hiPSC-CMs vs. Animal Models and Heterologous Expression System

Before developing hiPSC-CMs, it was useful to use immortalized cell lines overexpressing specific ion channels or transgenic mouse models to replicate LQTS *in vitro* and *in vivo* (10). However, the limitations are pretty obvious. Firstly, these cells cannot replicate the physiological environment of primary cardiomyocytes, which could result in false positives or negatives. For example, to model LQT2, the expression of the *KCNH2* gene encoding a potassium channel in HEK-293 cells alone seems less than ideal for its intracellular maturation and supracellular membrane function. The presentation of the hERG channel's full physiological state requires the auxiliary subunit minK to form a stable multimer, which is lacking in a heterologous expression system (17, 18). Co-expression of them in HEK-293 cells, on the other hand, requires consideration of the operation strategy of the researcher, such as differences in transfection ratios and their sensitivity to cardiotoxic drugs when present alone or together in a physiological context (19).

As for animal models, the implications of species differences in inconsistency in cardiac electrical activity need to be considered. Although ion channels are highly conserved in humans and mice, the substantial differences in gene expression profiles and physiology between species severely limit the validity of extrapolating human data from rodents. Such differences are more pronounced in mice, as evidenced by higher heart-rate differences and repolarization performance (20). And the low level of expression of channels mediating repolarizing currents limits the use of models in knockdown expression. Because of species differences in cardiac electrical function features, these different models do not accurately represent all aspects of the human disease (20).

hiPSC-CMs express a variety of ion currents essential for the heart, such as I_Na_, I_CaL_, I_to_, I_Kr_, and I_Ks_ (21). Although still immature, mainly reflected by the low expression of I_K1_ and the high expression of the pacemaker current, they are important for forming resting potentials, notably for the action potential (AP) generation and profile (22, 23). The fetal-like myocardial function may impair the accurate modeling of adult LQTS. For example, low expression of I_K1_ makes hiPSC-CMs dependent on I_Kr_ to obtain the maximum diastolic potential (MDP), which is markedly depolarized with I_Kr_ blockers, negatively affecting LQT2 disease replication and screening for hERG channel toxicity (23–26). These deficiencies are not negligible for the study of LQTS. Multiple cues to promote the maturation of hiPSC-CMs, including prolonged culture time, biophysical stimulation, and 3D culture, also help to address this (23). 3D Cardiac tissue shows longer AP duration (APD), hyperpolarized resting membrane potential, and faster upward velocity, with sodium current density and upward velocity similar to human ventricular tissue (27).

### Generation of hiPSC and Directed Cardiomyocyte Differentiation

**Figure 1:**
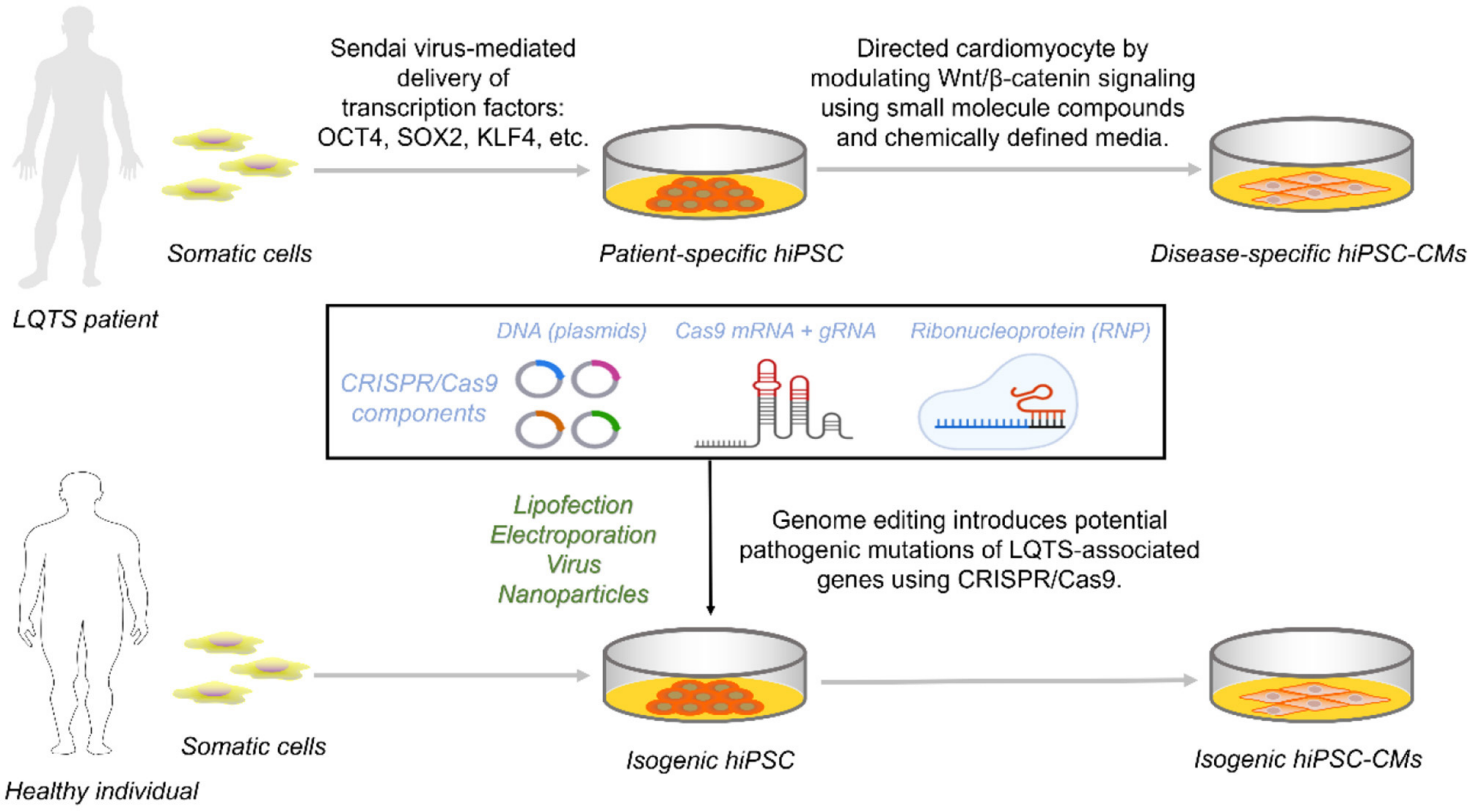
Access to disease-specific hiPSC-CMs from LQTS patients and isogenic cell lines by CRISPR/Cas9. Reprogramming somatic cells from healthy individuals or patients with transcription factors generates hiPSCs that are further targeted to inhibit Wnt/β-catenin capable of directed differentiation into hiPSC-CMs carrying the genetic information. For hiPSCs from healthy individuals, constructing an isogenic set as control requires the introduction of one of the three CRISPR/Cas9 components into the cell *via* a different vector.

Circumventing the ethical limitations of hESCs, reprogrammed pluripotent stem cells from somatic cells of LQTS patients are induced by four specific transcription factors (28–30). Transcription factors are mainly delivered into any cell type with proliferative potential by viral vector-based methods (retroviruses, lentiviruses, and inducible viruses) and non-viral vector methods (plasmids or linear DNA and transposons) (31). Initially, skin dermal fibroblasts were used, followed by non-invasive peripheral blood cells, including T and B cells, and even uroepithelial cells (32, 33). Retroviruses and lentiviruses, as delivery vectors, raise concerns about the risk of insertional mutations and even karyotypic abnormalities (34–36). The use of non-integrating viruses (e.g., Sendai virus), free vectors, or the formation of gene integration-free hiPSC *via* direct delivery of reprogramming factors (e.g., proteins or mRNA) has emerged as an excellent option (37–39) (Figure 1). Of course, each approach has its own set of benefits and drawbacks. And no reprogramming delivery mechanism has ever been modified without encountering severe restrictions or unintended consequences (40).

The development of hiPSC-CMs is designed to mimic the human mesoderm and heart development process (41). Briefly, TGF-β activates the typical Wnt/β-catenin pathway to signal initiation and subsequent mesoderm formation and is inhibited late in the cardiac lineage (42). Chemically defined media containing Wnt inhibitors promote mesoderm formation capable of efficient formation of cardiomyocytes (43, 44) (Figure 1).

LQTS individual-derived hiPSCs are directionally differentiated and retain their genetic background to form disease-specific hiPSC-CMs, giving most experimental platforms a novel approach to ICCs research. LQT1 was the first channelopathy to be modeled using hiPSC-CM. This study derived hiPSCs from two LQT1 patients carrying the genetic heterozygous missense mutation *KCNQ1*-R190Q. LQT1 hiPSC-CMs summarize the distinctive features of LQT1, including a reduction in AP repolarisation currents I_Ks_, leading to a prolongation of APD and a channel protein transport defect that exhibits a dominant-negative effect (45). Although it is not always possible to enroll LQTS patients in some laboratories, the development of CRISPR/Cas9 has alleviated this problem. Researchers can alter the genetic information of normal people or LQTS patients nearly at will by modifying the genome of hiPSC with CRISPR/Cas9 (46) (Figure 1). And the reproducibility of such genomic modifications in hiPSC is far superior to the manipulations performed in terminally differentiated hiPSC-CMs.

### Electrophysiological Characterization of LQTS-hiPSC-CMs

The patch-clamp technique is the gold standard for measuring various AP parameters from hiPSC-CM. They chiefly include resting membrane potential (RMP), MDP, and APD at different repolarization percentage levels (i.e., APD20, APD50, and APD90) that correspond to specific ion current characteristics (21). Researchers have focused more on APD for LQTS since APD90 is most dependent on I_Ks_ and I_Kr_ (9). Using a dynamic clamp characterized by the real-time evaluation and injection of simulated membrane current, the injection of I_K1_ into hiPSC-CMs to achieve a close-to-physiological RMP minimizes the LQTS model's inaccuracy (47–50).

Compared to the patch-clamp, no other technique can isolate individual sodium or potassium channels. However, this low-throughput and relatively complex technique is insufficient for academic and industrial laboratories. Despite the lower resolution, a high-throughput multi-electrode array (MEA) can obtain *ex vivo* cardiac field potential durations (FPD) correlated with QT interval characteristics in ECG and aid in the analysis of normal or prolonged APD (51–57).

Abnormal electrical activity, such as decreased I_kr_ in LQT2 and I_ks_ in LQT1, increased late sodium currents (I_NaL_) in LQT3, prolonged APD and FPD, and the occurrences of after early depolarizations (EADs), can be detected in the laboratory using these techniques in disease-specific or CRISPR/Cas9 editing-based LQTS models (Table 1). Different LQTS-associated SNVs can be assessed for drug response in hiPSC-CM using high-throughput electrical measurements and simple gene editing (53, 54, 108, 109). Moreover, optical evaluations by mapping the hiPSC-CMs with genetically encoded voltage and fluorescent calcium indicators fulfill the potential for studying conduction and arrhythmogenesis (110, 111). They are progressively identifying intracellular calcium transient abnormalities as an important mechanism of abnormal phenotypic in LQTS, which could lead to new therapeutic options (8).

**Table 1:** Disease-specific and CRISPR/Cas9-edited LQTS-associated variants characterized in hiPSC-CMs.

**Disease**	**Variants**	**Properties**	**Sources**	**Phenotype**	**References**
LQT1	*P*.308 ~ 344del, G589D, IVS7-2A>G, M437V, R190Q, S566Y, Arg401fs, G314S, A190G	Heterozygous	LQT1 patients	Lower I_Ks_ amplitude and prolonged APD (*P*.308 ~ 344del, M437V) Abnormal calcium transient (G589D, IVS7-2A>G) LUF7346 enhanced I_Ks_ (R190Q) Not performed (S566Y, Arg401fs, G314S, A190G)	(58–66)
	G179S	Homozygous	LQT1 patients	Not performed	(67, 68)
	R594Q	Homozygous (JLNS) Heterozygous (LQT1)	LQT1 and JLNS patients	LUF7346 enhanced I_Ks_	(62)
	A344Aspl	Synonymous	LQT1 patients	Prolonged cFPD	(69)
	R190Q, G269S, G345E	Heterozygous	ZFN-mediated targeted gene	Prolonged APD	(70)
	Y171X, V254M, I567S, A344A/spl	Heterozygous	LQT1 patients, CRISPR/Cas9 (A344A/spl)	Prolonged APD	(71, 72)
	c.569 G>A, c.585delG, c.573_577delGCGCT	Unavailable	LQT1 patients	Not performed	(66)
LQT2	G1681A, R176W, A561T, A561V, A561P, L1012P, N996I, IVS9-28A/G, A422T, G604S, N633S, R685P, V822M, P605L, T152P, R366X, S428X, c.1714G>A, c.1870A> T, c.2960del, R752W	Heterozygous Variant of uncertain significance (c.1870A>T)	LQT2 patients Individual carrying the variant (c.1870A>T)	Prolonged cFPD and EADs (G1681A) Lower I_Kr_ amplitude and prolonged APD (R176W, A561T, A561V, A561P, N996I, IVS9-28A/G, G604S, N633S, R685P, V822M) Not performed (L1012P, P605L, T152P, c.1714G>A, c.1870A> T, c.2960del) Lower I_Kr_ amplitude and greater I_CaL_ (R752W) Prolonged cFPD and abnormal calcium transient (A422T, A561V, IVS9-28A/G, R366X, S428X)	(8, 56, 62, 73–86)
	G603D, c.1841C > T, c.2464G > A	Unavailable	LQT2 patients	Unavailable (G603D) Not performed (c.1841C > T, c.2464G > A)	(87, 88)
	A614V	Heterozygous	ZFN-mediated targeted gene	Prolonged APD	(70)
	A422T, G601S, R534C	Heterozygous	LQT2 patients, CRISPR/Cas9	Prolonged APD and abnormal calcium transient (A422T, G601S) Lower I_Kr_ amplitude and prolonged APD (R534C)	(72, 89, 90)
	T983I	Variant of uncertain significance	Individual carrying the variant	Lower I_Kr_ amplitude and prolonged APD	(91)
	A561T, N996I, K897T, A561V	Heterozygous	CRISPR/Cas9	Lower I_Kr_ amplitude	(50, 92, 93)
	p.S1112Pfs*171	Frame-shift variant	LQT2 patients, CRISPR/Cas9	Prolonged cFPD and APD	(16)
LQT3	V1763M, R535Q, V240M, N406K, E1784K, R1644H, R1623Q, N1774D, D1275N, 1795insD+/-	Heterozygous	LQT3 patients CRISPR/Cas9 (N406K)	Prolonged APD and increased I_Na_ (V1763M, R1623Q, N1774D) Longer inactivation of I_Na_ (R535Q, V240M) Reduced I_Na_ (E1784K, N406K) Prolonged cFPD (R1644H) Reduced I_Na_ (D1275N) GS967 inhibited I_Na_ (1795insD+/-)	(72, 90, 94–101)
	W156X, R1638X	Nonsense mutation	LQT3 patients	Reduced I_Na_ and AP upstroke velocities	(102)
	F1473C, N406K	Unavailable	LQT3 patients	Prolonged APD and increased I_Na_ (F1473C) Abnormal calcium transient (N406K)	(103, 104)
	S1103Y, R1193Q	Heterozygous Homozygous	LQT3 patients, CRISPR/Cas9	Prolonged APD (S1103Y) Increased I_Na_ and I_Kr_ (S1103Y, R1193Q)	(105–107)

## RNA-Guided Gene Modification in hiPSC

Earlier, various single-gene modification efforts were performed in hiPSC using genome editing techniques such as zinc finger nuclease (ZFN) and transcription activator-like effector nuclease (TALEN) (112). ZFN-driven transgene addition of *KCNQ1* and *KCNH2* has successfully modeled the disease (70). Unlike ZFN and TALEN, which use interactions between amino acid residues and nucleotides to identify DNA target sites and induce double-strand breaks (DSBs), CRISPR/Cas9 uses absolutely specific nucleotide-nucleotide base pairing to make it a more efficient and precise gene-editing tool (113, 114). Genome editing based on CRISPR/Cas9 has undoubtedly become the primary means of modifying the expression of specific genes at the hiPSC. Off-target mutations in hiPSCs may be sufficiently low to be a non-issue in disease modeling and other applications (115).

**Figure 2:**
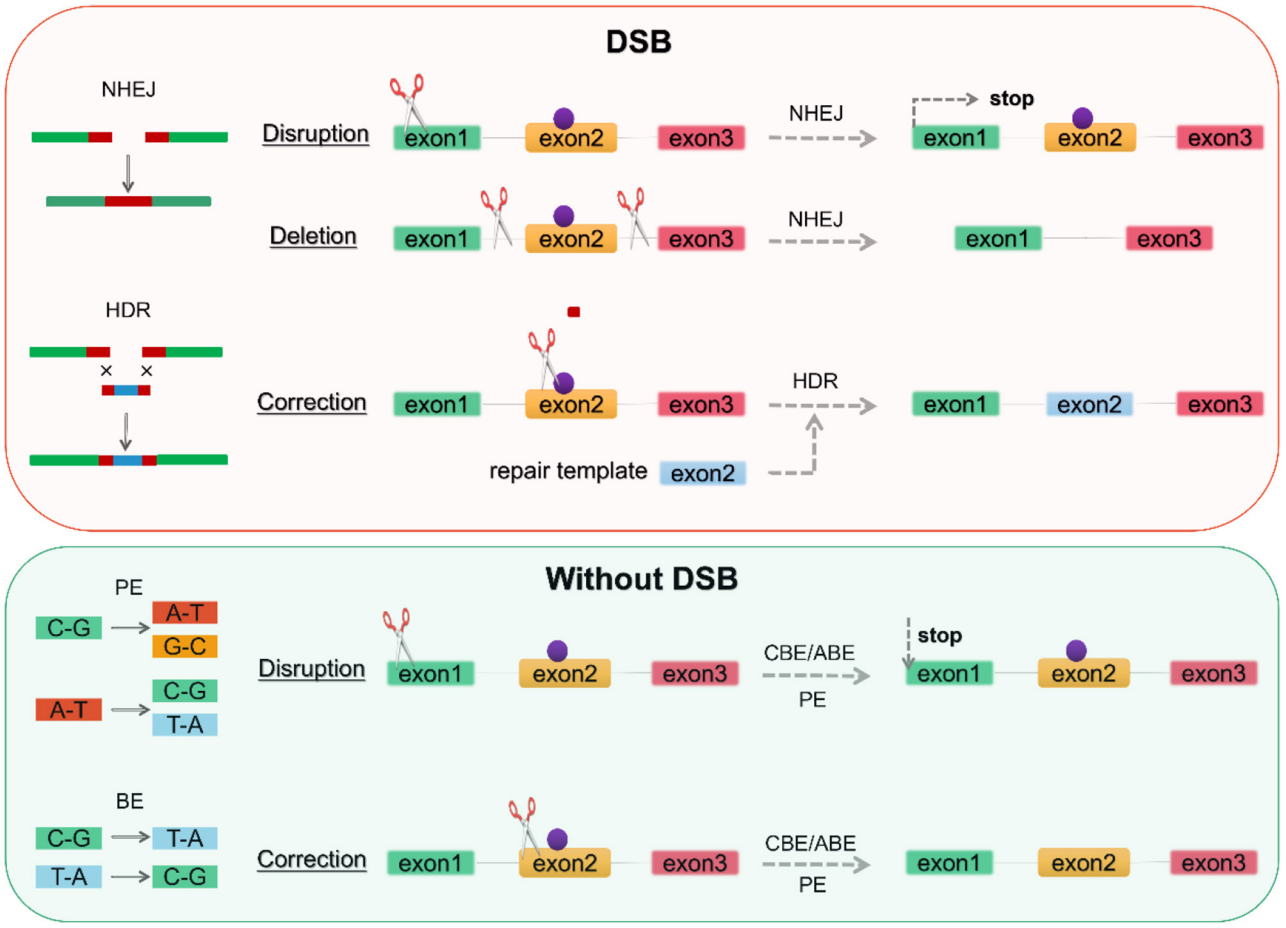
Genome editing according to repair mechanism. DSBs induce two repair mechanisms, NHEJ and HDR. NHEJ can generate an indel at the gene of interest-based on gene disruption resulting in premature translation stop to achieve knockout, while gene deletion requires the creation of two DSBs on both sides of the pathogenic mutation (purple point). BE and PE can perform base substitution without DSBs, with the former being limited to 8 base substitutions and the latter enabling arbitrary base substitutions. DSB, double-strand breaks; NHEJ, non-homologous end joining; HDR, homologous directed repair; BE, base editor; PE, prime editor. CBE, cytosine base editor; ABE, adenine base editor.

The CRISPR/Cas9 system consists of **two** components, CRISPR and Cas9. Two repair mechanisms of Cas9-induced DNA DSBs, non-homologous end joining (NHEJ) or homologous directed repair (HDR), are utilized for the alteration of genomic DNA sequences to prevent or treat a disease: disruption of a gene, deletion of a specific genomic region, and correction of a gene (Figure 2). CRISPR can usually be designed and substituted as a single guide RNA (sgRNA or gRNA) at LQTS-associated gene target sites (116, 117). CRISPR/Cas9 cargoes can be of three types, namely (1) DNA plasmid encoding both the Cas9 protein and the guide RNA, (2) a combination of sgRNA and Cas9 mRNA, and (3) Cas9 protein with gRNA (ribonucleoprotein complex, RNP) (118–120) (Figure 1). Furthermore, both gene correction and gene addition require an exogenous single- or double-stranded DNA (ssDNA) template with homologous arms or a single-stranded DNA oligonucleotide (ssODN) (46, 113). For example, SNV for *KCNH2*, isogenic correction ssODN templates need to contain wild nucleotide sequences and CRISPR/Cas-blocking mutations, preferably in the PAM, to minimize undesirable re-editing (91, 121) (**Figure 4**).

Provided that a donor template is available, DSB-mediated HDR repair mechanisms allow the introduction of targeted mutations associated with the LQTS phenotype or the correction of pathogenic mutations. Nevertheless, in addition to consideration of off-target, DSB leads to activation of the p53 pathway inducing DNA damage responses and cell cycle arrest (122, 123). And genome editing by CRISPR/Cas9-induced DSBs is generally less efficient in hiPSCs compared to 293T cells (124, 125). DNA base editors (BEs), including the cytosine base editor (CBE) and adenine base editor (ABE), have been proposed to perform precise nucleotide substitution without the need for a donor template or the introduction of DSBs (46, 126–128) (Figure 2). Prime editors (PEs) extend the limited editing window of BEs (C-G to T-A conversion for CBE and A-T to G-C conversion for ABE) to **eight** transition mutations (C→ A, C→ G, G→ C, G→ T, A→ C, A→ T, T→ A, and T→ G) (129–132) (Figure 2). PEs substantially expands the scope and capabilities of genome editing and, in principle, could correct up to 89% of known genetic variants associated with human diseases.

## Deciphering LQTS Using CRISPR/Cas9 and hiPSC-CMs

### Generation of Isogenic Sets of hiPSC-CMs Using CRISPR/Cas9

Clinical genetic testing has become the standard for diagnosing genetic variants in suspected monogenic disorders. However, these results are often found for VUS, for which they do not have sufficient evidence of pathogenicity. VUS is becoming a significant challenge in clinical genetics. The critical issue remains that the variable expression and incomplete penetrance between individuals with the same LQTS pathogenic mutation remain largely unexplained (7). As a result of the progressive use of genetic testing, the discovery of more rare variants, modifier genes with clear variants, and VUS, combined with the lack of experimental platforms, the focus has become on how to rapidly and reliably determine the functional significance of the genetics of variants.

*In vitro* hiPSC-CMs models of LQTS can be derived from disease-specific somatic cell differentiation and differentiation of CRISPR/Cas9-edited hiPSCs. The main reason for generating these hiPSC-CMs is to observe the effects of mutations on cell phenotypes, particularly genome editing using CRISPR/Cas9. It is worth considering that multiple genetic and epigenetic differences exist between cell lines and that reprogramming and genome editing are themselves the causes of epigenetic variation. Moreover, genetic background variation may confound disease characteristics, particularly for LQTS with incomplete penetrances (73). It requires the generation of appropriate control cell lines (isogenic cell lines) with the same genetic background (Figure 1). Isogenic hiPSCs provide reliable and effective model alternatives for human disease research (50, 73, 89, 91, 133).

For disease-specific differentiation of hiPSC, unrelated healthy individuals need to be recruited to generate hiPSC-CMs under the same conditions. CRISPR/Cas9-edited hiPSCs can then insert the same mutations into control hiPSC lines or introduce disease-causing variants in normal hiPSCs (Figure 1). Even comparing LQTS-specific to genome-edited isogenic cell lines is a strategy that could be considered. It could be an attempt to discern their superiority or inferiority in LQTS modeling by eliminating genetic background interference (89). In terms of these considerations, disease-specific hiPSC-CMs may have an advantage in overcoming the detrimental effects, such as p53 pathway-induced cell apoptosis associated with genome editing. Subsequently, CRISPR/Cas9 corrective mutations performed in the same genetic background from the same individuals to produce isogenic control sets would provide stronger evidence for the reversal of abnormal cell phenotypes than control sets from unrelated individuals. Unfortunately, sometimes, such disease-specific individual patients are not easily recruited, especially those with rare variants, which impedes the timely and effective management of those patients carrying what is defined as VUS after the genetic screening. Due to its high efficiency, CRISPR/Cas9 is also a powerful tool for establishing homogeneous controls when outlining the disease profile of LQTS.

#### LQT1

**Figure 3:**
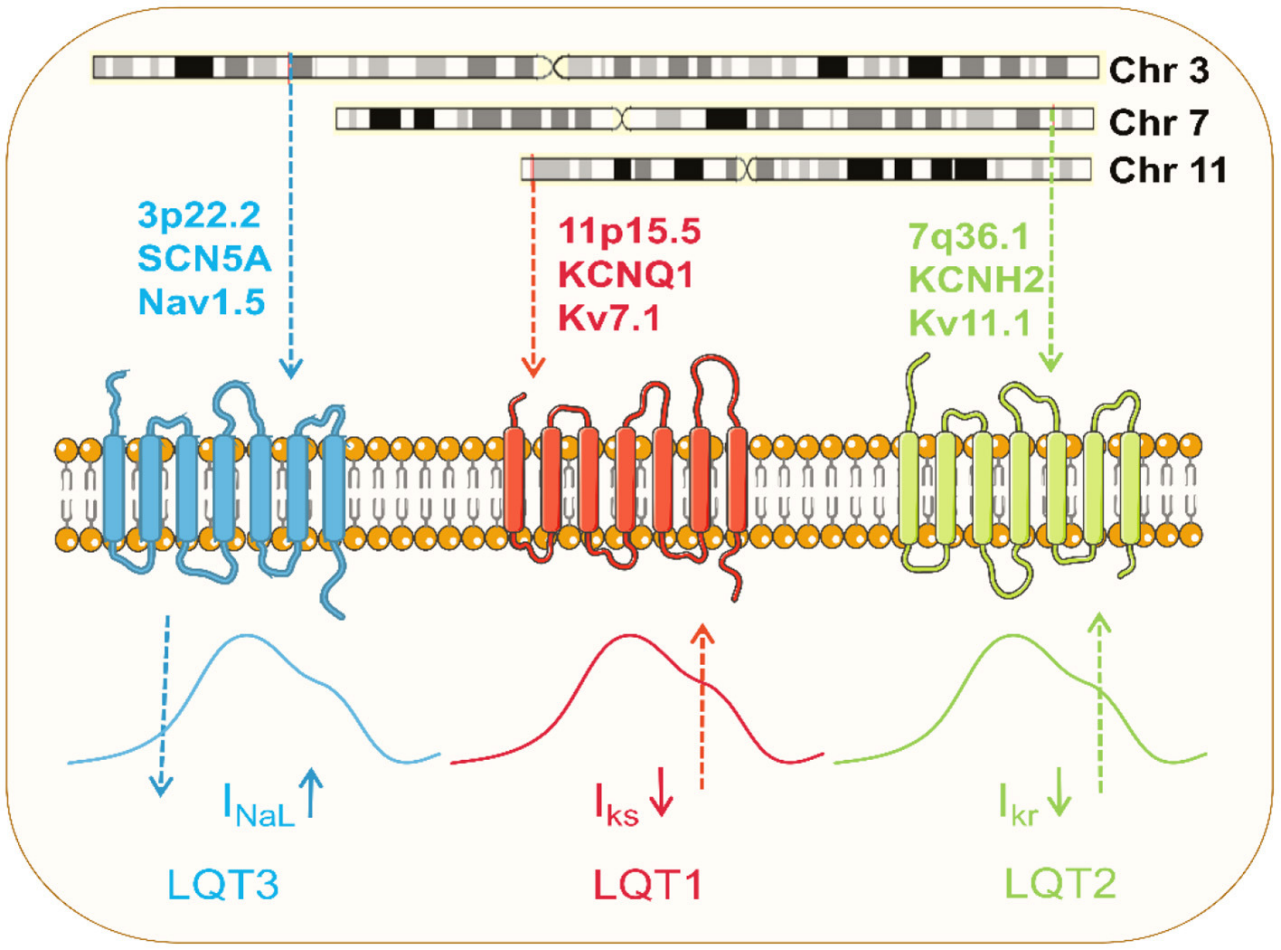
Pathophysiology of the common LQTS. Dominant mutations in *KCNQ1* on chromosome 11 and *KCNH2* on chromosome 7 result in loss of function of Kv7.1 and Kv11.1 potassium channels, respectively, and these channel currents play a significant role in myocardial repolarization. Pathogenic mutation in *SCN5A* on chromosome 3 causes gain of function of Nav1.5 channel, resulting in increased late sodium currents to prolong the action potential.

In 1996, *KCNQ1* was identified as the gene responsible for LQT1 (134). LOF mutations in *KCNQ1* to reduce I_Ks_ are the most common cause of congenital LQTS, occurring in 40–50% of all patients, and are referred to as LQT1 (135) (Figure 3). Numerous models of LQT1 hiPSC-CMs carrying SNVs are summarized in Table 1.

Before applying genome editing in hiPSC, controls were generated based on unrelated healthy individuals with hiPSC-CMs. The generation of isogenic cells is not only salutary for modeling but also for explaining clinical heterogeneity (13). At this point, the introduction of CRISPR/Cas9 can target not only *SCN5A* itself but also *SCN5A* modifiers, such as myotubularin-related protein 4 (MTMR4) (13). The impact of an indirect generation of wild or mutated modifiers on the phenotype of isogenic cell lines identifies the underlying regulation of *SCN5A* to produce diverse clinical manifestations. Their recognition can improve risk stratification and clinical management. And even though mutation correction is inefficient and unavailable for mature heart cells, the CRISPR/Cas9 system remains a powerful tool for validating its gene therapy potential by generating normal isogenic controls (14, 71).

#### LQT2

LQT2 is the second most common LQTS and results from mutations in the *KCNH2*, also known as the hERG gene (136). Dominant mutations present a haplotype deficiency or dominant-negative effect, causing a partial or complete reduction in I_Kr_ current and prolonging APD (137) (Figure 3). Modeling hiPSC-CMs avoids the problem of changes in channel activity generated by the absence of auxiliary subunits. I_Kr_ has been reported in hiPSC-CM with a current density value comparable to native human myocardium (10). Like LQT1, several SNVs in LQT2-hiPSC-CMs have been developed (Table 1). Itzhaki et al. first established a model containing the *KCNH2*-A614V mutation using dermal fibroblasts from LQT2 patients. APD and FPD were significantly prolonged compared to healthy control models (138).

The isogenic set generated by CRISPR/Cas9 editing makes it convenient to replicate the newly identified LQTS variants in hiPSC-CMs. More recently, in a genetically elusive multigenerational LQTS pedigree, a frameshift variant (p.S1112Pfs*171) in patient-specific hiPSC-CMs was identified as a novel LQT2-causative variant to induce prolonged FPD, a result compared to CRISPR/Cas9-corrected isogenic control hiPSC-CMs (16). Besides, as hERG channels are the most common targets of drug-acquired LQTS, modeling using hiPSC-CMs combined with high-throughput assays of MEA offers great advantages in assessing multiple groups of cardiotoxic drugs (53, 54, 139). More importantly, the genetic background may influence individual sensitivity to these drugs (19, 55, 72, 73). Through introducing different variants in healthy wild-type hiPSC without any known disease-causing mutations, isogenic sets of hiPSC-CMs with distinct *KCNH2* mutations differ functionally and in susceptibility to drug-induced arrhythmias (50, 72, 89, 91). Targeted editing of *KCNH2* using CRISPR/Cas9 to generate hiPSC-CMs carrying SNPs supports hiPSC-CMs as solid candidates for evaluating the underlying severity of *KCNH2* mutations, which could facilitate patient risk stratification.

#### LQT3

LQT3 is caused exclusively by a gain-of-function mutation in the *SCN5A* gene that encodes for the alpha subunit of the Nav1.5 (94, 140). *SCN5A* mutations cause gain-of-function of Nav1.5 channels by impairing channel inactivation and accelerating recovery from inactivation, increasing I_NaL_ to counter repolarization and prolonging APD (103) (Figure 3; Table 1).

The primary question for simulations of the *SCN5A* mutation may be the relationship between genotype-phenotype (141). Mutations are associated with several genetically heterogeneous disorders, including Brugada syndrome (BrS), cardiac conduction disease (CCD), sick sinus syndrome (SSS), and others (142). Genotype-phenotype studies in large pedigrees have established that several single *SCN5A* mutations present with multiple clinical manifestations due to the various biophysical defects.

CRISPR/Cas9 generation knockdown of *SCN5A* to generate the Nav1.5 KO hiPSC lineage or the introduction of pathogenic mutations to build control models has great breakthroughs in analyzing the pathophysiological mechanisms of the phenotype (15, 105, 106). Similarly, drug arrhythmogenic susceptibility can be determined using genomically corrected *SCN5A* variant isogenic control cell lines (105). Even introducing CRISPR/Cas9 in hiPSCs makes it possible to compare the properties of different variants, a single variant in different cell models, or even differences between homozygous or heterozygous mutation (107, 143).

### Elucidation of Gene Modifiers and VUS

#### LQT1

Modifier genes are genes at the same or other loci that affect the phenotype of the major gene (144). Non-synonymous coding variants common to the same major mutant gene and common variants in genes encoding ion channel regulators or auxiliary subunits can also act as gene modifiers (144). *KCNE1* encoding the Kv7.1 auxiliary subunit (p.Asp85Asn, also known as D85N) is more susceptible to LQTS and may be associated with an increase in disease severity (145). These observations can be explained by impaired repolarisation currents (I_Ks_, I_Kr_) when this *KCNE1* variant interacts with *KCNQ1* or hERG channels. Non-coding elements required for mRNA stability and translation exist in the genes' 3′-untranslated region (3′-UTR). Variants in the 3′-UTR are only related to a prolonged QT interval in heterozygous *KCNQ1* mutations, not in the general population (146, 147). Conversely, the SNVs of the gene may also be an independent modifier to confer protection against cardiac events in patients with LQTS (13, 148).

It is helpful to explain monogenic diseases' incomplete penetration and variable expression. Further examples include AKAP9 and NOS1AP variants (3, 149–151). Recently, two SNVs on the myotubularin-related protein 4 (MTMR4) gene were identified by whole-exome sequencing as potential contributors to the clinical phenotype of LQT1 patients (13). As an interactor of Nedd4L, the MTMR4 variant is thought to be responsible for the different clinical manifestations of members of the same family carrying the same mutation, *KCNQ1*-Y111C. It was discovered that hiPSC-CMs from asymptomatic patients showed increased degradation of *KCNQ1* than hiPSC-CMs from symptomatic patients, who showed reduced degradation of *KCNQ1* and hERG proteins due to reduced Nedd4L activity caused by the MTMR4 variant. And the correction of SNVs in MTMR4 by CRISPR/Cas9 unmasked the LQTS phenotype (13). While new modifier genes are proposed, it is worth testing whether such protective modifications are present in more monogenic diseases, as in the case of *KCNQ1* polymorphism rs2074238 T-allele (148).

Given the above, a conclusion can also be drawn: if no disease phenotype is observed, this does not necessarily mean that the variant is benign. The disease phenotype may be masked if the mutation has low penetration and the control hiPSC line selected carries a protective genetic modifier. In such cases, it is essential to correct for these protective SNVs in the patient-derived hiPSC to demonstrate their effect on the disease phenotype.

#### LQT2

As with LQT1, modifier genes explain the clinical genotype-phenotype inconsistency of LQT2. It is important to investigate this issue by examining modifier genes, introducing them to hiPSC-CMs, and characterizing their function. *KCNH2*-K897T is the most commonly reported genetic modifier of *KCNH2*, which impairs the repolarisation reserve associated with LQT1 and LQT2 mutations, and exacerbates LQTS (144, 152–155). For example, K897T with the *KCNH2* mutation (A1116V) exacerbates the reduction in I_Kr_ caused by the latter to produce symptoms, whereas there are no symptoms when A1116V is present alone (154).

Conversely, some protective mutations like *KCNH2*-R1135H maintain I_Kr_ channel function (156). Isogenic hiPSC-CMs lines with K897T in cis and trans were produced using CRISPR/Cas9 to see if the linkage phase of the K897T polymorphism to the major *KCNH2* polymorphisms A561T and N996I caused changes in I_Kr_ (92). The findings show that the common polymorphism *KCNH2*-K897T has a different effect on LQT2-causing *KCNH2* mutations depending on whether it is present in cis or trans (92). Similarly, the correction of K897T variants with CRISPR/Cas9 would be valuable in determining whether this common polymorphism plays a protective or aggravating role in QTc prolongation and the corresponding effect on disease severity. Genome sequencing provides more evidence of genetic modifiers to explain genotype-phenotype discordance (74, 157, 158). In the LQT2 lineage, genomics identified **two** modifier genes, *KCNK17* and *REM2*, to elucidate the contributors to variable expressivity of *KCNH2*-R752W mutation (74). CRISPR/Cas9 editing to correct a *REM2* variant reversed the enhanced I_CaL_ and prolonged APD observed in hiPSC-CMs from severely affected individuals (74).

**Figure 4:**
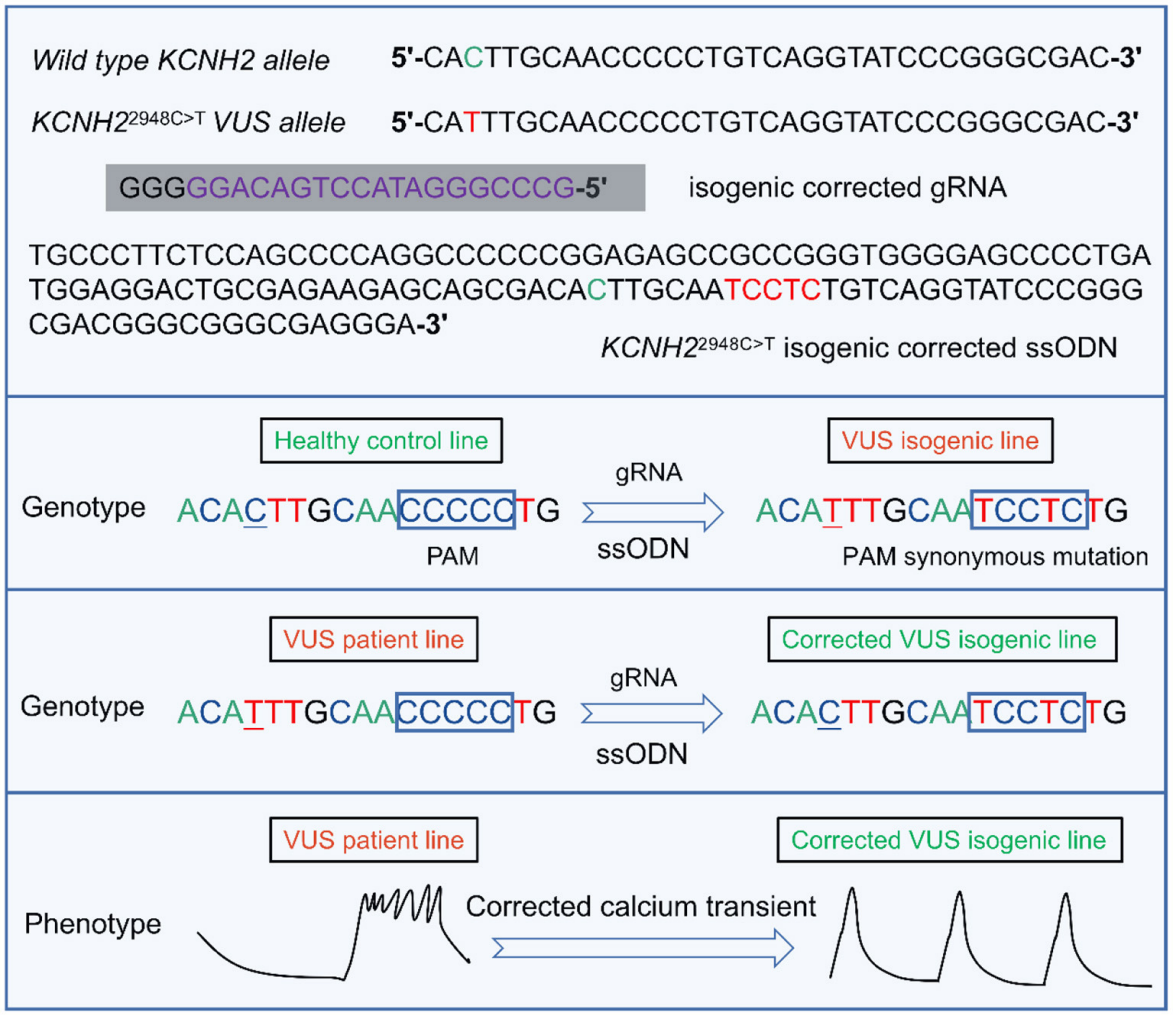
A representative example of the targeted design of isogenic corrected gRNA or ssODN for the locus where *KCNH*2^2948C>T^ VUS is located, enabling correction of the point mutation (91). gRNA is designed by targeting the upstream of the 5'-NGG (PAM) of the *KCNH2* VUS locus. By introducing ssODN containing homologous arms, the VUS isogenic line can be generated in a healthy control cell line. Similarly, the VUS line can also be corrected to enable a phenotype of abnormal calcium transient to be reversed. VUS, variants of uncertain significance. PAM, protospacer adjacent motif. gRNA, guide RNA. ssODN, single-stranded DNA oligonucleotide.

Another study of interest is the only **one** to date to characterize *KCNH2*-T983I (*KCNH*2^2948C>T^) VUS in LQTS using hiPSC-CMs combined with CRISPR/Cas9 editing (91) (Figure 4). Determining the pathogenicity of a VUS is challenging due to the lack of suitable model systems and accessible technologies (12). Approximately 40% of variants are designated as still not classifiable as pathogenic or benign (159). Multiple family members carrying the same mutation may have different QT intervals and clinical presentations. Moreover, in the era of next-generation sequencing, the interpretation of the increasing number of identified unknown variants will pose an even greater therapeutic challenge. Thus, Garg et al.'s research was groundbreaking, demonstrating that genome editing in hiPSC could be a helpful tool for determining the pathogenicity of VUS in cardiac channelopathies (91). They generated hiPSC-CMs from peripheral blood mononuclear cells carrying the novel VUS mutation *KCNH2*-T983I. VUS hiPSC-CMs exhibited significantly prolonged APD, reduced I_Kr_, and abnormal calcium transients compared to a healthy control line. Further correction of VUS hiPSC-CMs with CRISPR/Cas9 resulted in a normal phenotype, and the introduction of VUS in healthy hiPSC-CMs recapitulated the hallmark features of LQTS disease (91) (Figure 4). These attempts were able to categorize and manage VUS in a timely and effective manner without the development of SCD.

Identifying these variants can guide genotype-specific management or facilitate rapid screening of potentially high-risk relatives. Although distinguishing pathogenic from benign is a significant challenge, primarily when classified as VUS, this new approach still represents a significant advance in precision medicine for the management of LQTS disease (157).

#### LQT3

A modifier gene can also explain the phenotype caused by the SNVs in *SCN5A*. *SCN3B* encoding the Nav1.5 β-subunit amplifies the reduced sodium current generated by mutation *SCN5A*-E1784K and thus masks the Brs phenotype (95). Compared to the almost 100% penetration of the typical ΔKPQ variant initially described, the R1193Q is close to zero (107, 160). The prevalence of this variant in an unaffected population (6.1% in the East Asian allele) strongly suggests that it cannot play an essential role in disease manifestion (161).

The underlying mechanisms of *SCN5A* ΔKPQ and R1193Q-inducible variants were assessed by designing gRNAs to introduce R1193Q into *SCN5A* exon 19 of hiPSC from healthy individuals (107). The study suggests that the low penetration rate of the R1193Q mutation involves PI3Kα-mediated changes in PIP3-modulated I_NaL_. Unlike ΔKPQ, the sensitivity of R1193Q to PIP3 means distinct pathologies need to be considered when interpreting the severity of the excess late current functional defect in Nav1.5 (107). The observation of late current in an *in vitro* setting does not necessarily translate into a highly pathogenic LQT3 phenotype but rather depends on the underlying mechanisms. Nav1.5 channels differ in hiPSC-CMs of LQTS, or Brs may rely on different Tbx5 variants, such that LQT3 may be due to the failure of the Tbx5-D111Y mutation to repress CAMK2D and SPTBN4, which significantly enhances I_NaL_ (162).

### Potential Causative Genetic Modification Strategies

Attempts at effective management strategies in disease-specific hiPSC-CMs are valuable. The corrective effect of applying CRISPR/Cas9 against the SNVs of *KCNQ1* was **first** demonstrated in a hiPSC-CMs carrying a double mutation (c. 605-2A>G and c. 815G>A). Splicing mutations causing exon skipping lead to significantly longer APD90 and EADs; designing gRNAs to target these SNVs can correct aberrant cellular phenotypes (14). Nevertheless, unable to rescue two mutations simultaneously by constructing a CRISPR/Cas9 system containing two guide RNAs. In LQT1, *KCNQ1* variants often exhibit dominant-negative effects (163–167). Therefore, substitution alone, such as silencing the mutant allele using RNA interference (RNAi) without affecting the wild allele, is not sufficient; such a strategy requires that the effect of silencing exceeds that of wild-type expression, and the best outcome is to present a haplotype deficiency. Attempts to replace the mutant allele are feasible, and targeting SNV in CRISPR/Cas9 to design a segment of ssODN as a template for repair is feasible. The recently reported strategy of a dual-component suppression-and-replacement (SupRep) *KCNQ1* gene therapy targeting two variants of *KCNQ1* is also a clever optional strategy (71). Unfortunately, all of these tools face the problem of how to push data to the living body for the next validation step.

As previously stated, allele-specific RNAi was developed with targeted mutant KD to eliminate dominant-negative interference, assuming that the remaining WT allele would provide enough function to alleviate the disease phenotype despite haploinsufficiency. In previous studies of LQTS, allele-specific small interfering RNAs rescued the hERG current in heterologous expression systems through specific KD of the dominant-negative missense variants *KCNH2*-E637K (168). Such experiments in hiPSC-CMs were also victorious against *KCNH2*-G1681A (75). However, the end of the haplotype deficiency generated by dominant-negative effect RNAi appears promising. However, its implementation is still limited, such as the difficulty of designing each RNAi targeting SNVs and the fact that designing separate RNAi targeting specific individual variants is impractical when multiple variants are present simultaneously (71).

The advent of BEs and PEs seems to have made the correction of SNVs easier. But CRISPR/Cas9 still has a high probability of off-targeting associated with spatially inhomogeneous tolerances for pairwise mismatches in sgRNA-DNA heteroduplexes. They are still some way from being studied and applied *in vivo* (169). The most significant breakthrough in LQT2 treatment came with identifying the chemical chaperone lumacaftor, a drug approved by the FDA for the treatment of cystic fibrosis (8). Lumacaftor corrected two mutations, *KCNH2*-IVS9-28A/G and -A561V, representing trafficking defects in patient-specific hiPSC-CMs (8). Subsequent clinical translations showed good therapeutic effects in LQT2 patients (170). Targeting more *KCNH2* variants, lumacaftor had opposite effects in different cellular models (76, 171–173). They suggest that the dominance of the hiPSC-CMs model is responsible for the role of lumacaftor, which further delineates the differences between hiPSC and heterologous expression systems. Currently, a total of 160 *KCNH2* mutations representing protein trafficking defects are presented in the Milan database tested using Orkambi (lumacaftor plus ivacaftor) (7). This is another grand attempt by researchers to use the hiPSC-based platform to move toward precision medicine.

## Challenge and Perspective

After a decade of development, from the initial use of hiPSC to building a disease model to understand the clinical heterogeneity of the disease today combined with CRISPR/Cas9, the researchers have made a great deal of work. To achieve this, a large number of issues including consistently available *in vitro* research models, avoidance of ethical requirements and immune rejection, safety and efficiency of the reprogramming process, feasibility and efficiency of targeted differentiation, characterization of successful myocardial differentiation, off-target and cell safety of gene editing, quality control of epigenetics, and *ex vivo* and *in vitro* dissemination of the strategy, are being progressively addressed.

From somatic cells to hiPSC-CMs, the development of genome editing gives us more options in elucidating diseases. Experts in every field are working tirelessly to achieve the goal of precision medicine. In the case of a monogenic ICC such as LQTS, genome sequencing of individuals, and ongoing genome-wide association studies, identifies many possible variants that could be involved but need to be validated.

We need to figure out why people with the same SNV have varied clinical features, but we also need to test potential therapeutic options. Meanwhile, because there is a lack of VUS knowledge compared to typical dominant mutations, it is even more crucial to open a window of opportunity for timely intervention for patients carrying VUS or rare variants. Finally, achieving precision medicine may require collaboration from experts in clinical cardiology, electrophysiology, stem cells, genomics, and even pharmacology.

## Author Contributions

YS and ZZ: conceptualization, data curation, writing—original draft, software, and resources. JL: conceptualization, writing—review and editing, and supervision. All authors contributed to the article and approved the submitted version.

## Funding

This work was supported by the National Natural Science Foundation of Ningbo [Grant Number 2021J296] and the Natural Science Foundation of the Zhejiang Province [Grant Numbers LQQ20H160001 and LY21H020001].

## Conflict of Interest

The authors declare that the research was conducted in the absence of any commercial or financial relationships that could be construed as a potential conflict of interest.

## Publisher's Note

All claims expressed in this article are solely those of the authors and do not necessarily represent those of their affiliated organizations, or those of the publisher, the editors and the reviewers. Any product that may be evaluated in this article, or claim that may be made by its manufacturer, is not guaranteed or endorsed by the publisher.
