# Supplementary figures and images for: Deciphering Common Long QT Syndrome Using CRISPR/Cas9 in Human-Induced Pluripotent Stem Cell-Derived Cardiomyocytes
Source: Front Cardiovasc Med. 2022 May 13;9:889519. doi: 10.3389/fcvm.2022.889519 (PMC9136094; doi:10.3389/fcvm.2022.889519)

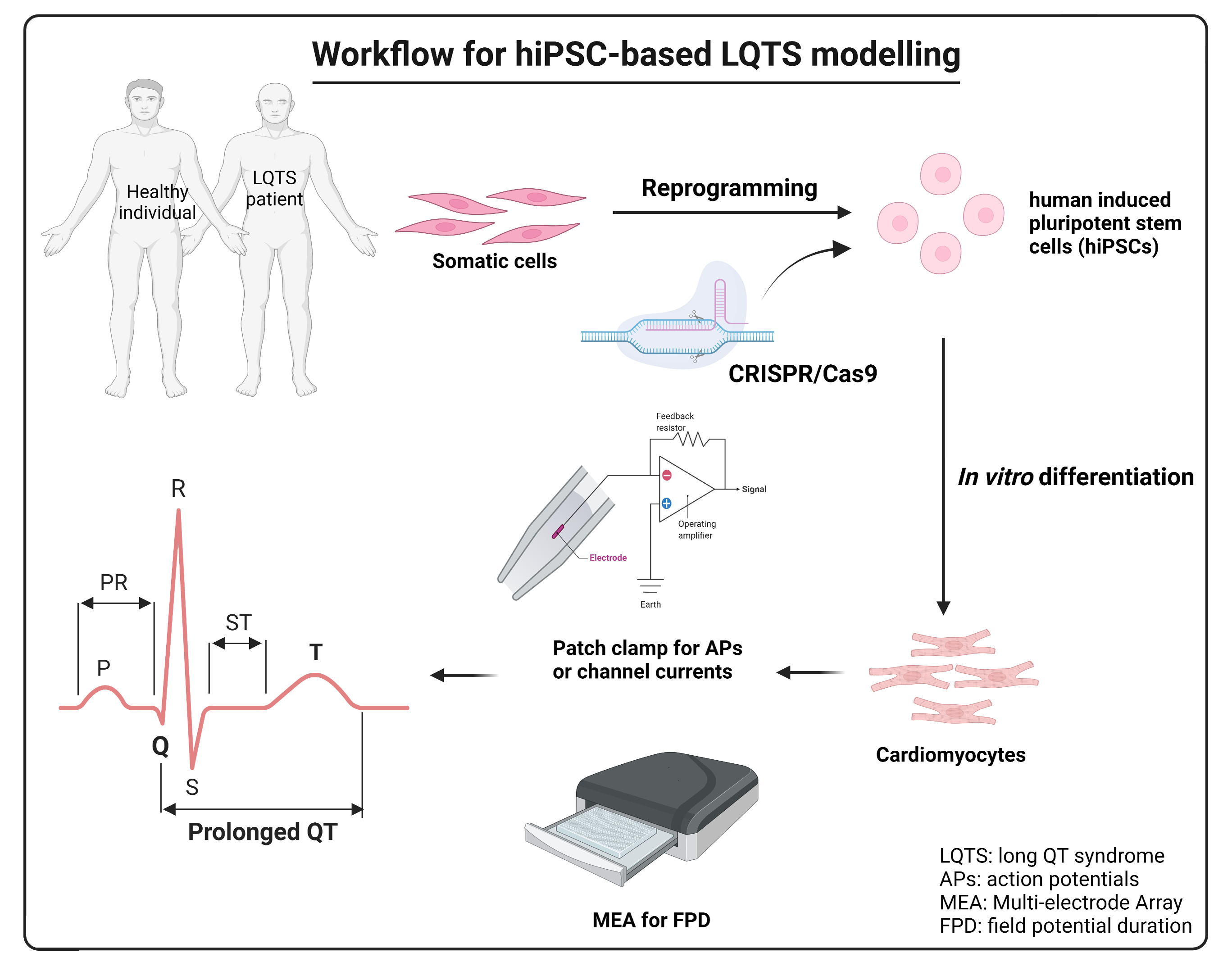

Supplement: Supplementary file 1 [file Image_1.PNG]
